# Genome-wide association studies in tropical maize germplasm reveal novel and known genomic regions for resistance to Northern corn leaf blight

**DOI:** 10.1038/s41598-020-78928-5

**Published:** 2020-12-15

**Authors:** Zerka Rashid, Mehrajuddin Sofi, Sharanappa I. Harlapur, Rajashekhar M. Kachapur, Zahoor Ahmed Dar, Pradeep Kumar Singh, Pervez Haider Zaidi, Bindiganavile Sampath Vivek, Sudha Krishnan Nair

**Affiliations:** 1 International Maize and Wheat Improvement Center (CIMMYT), ICRISAT Campus, Patancheru, Greater Hyderabad Telangana 502324 India; 2 High Mountain Arid Agricultural Research Institute (HMAARI) Stakna, SKUAST-Kashmir, Leh, 194101 India; 3 University of Agricultural Sciences, Krishi Nagar, Dharwad, Karnataka 580005 India; 4 Sher-E-Kashmir University of Agriculture Sciences and Technology (SKUAST), Srinagar, Jammu and Kashmir 190001 India

**Keywords:** Genetics, Plant sciences

## Abstract

Northern Corn Leaf Blight (NCLB) caused by *Setosphaeria turcica*, is one of the most important diseases of maize world-wide, and one of the major reasons behind yield losses in maize crop in Asia. In the present investigation, a high-resolution genome wide association study (GWAS) was conducted for NCLB resistance in three association mapping panels, predominantly consisting of tropical lines adapted to different agro-ecologies. These panels were phenotyped for disease severity across three locations with high disease prevalence in India. High density SNPs from Genotyping-by-sequencing were used in GWAS, after controlling for population structure and kinship matrices, based on single locus mixed linear model (MLM). Twenty-two SNPs were identified, that revealed a significant association with NCLB in the three mapping panels. Haplotype regression analysis revealed association of 17 significant haplotypes at FDR ≤ 0.05, with two common haplotypes across three maize panels. Several of the significantly associated SNPs/haplotypes were found to be co-located in chromosomal bins previously reported for major genes like *Ht2*, *Ht3* and *Htn1* and QTL for NCLB resistance and multiple foliar disease resistance. Phenotypic variance explained by these significant SNPs/haplotypes ranged from low to moderate, suggesting a breeding strategy of combining multiple resistance alleles towards resistance for NCLB.

## Introduction

Maize is the world’s leading cereal crop in terms of production, with 1016 million metric tons (MMT) produced on 184 million hectares (M ha) globally^1^, across tropical and temperate zones. About 80 per cent of the tropical maize is grown under rainfed conditions in sub-Saharan Africa, South and Southeast Asia, and Latin America, and is particularly vulnerable to an array of abiotic and biotic stresses. Among the biotic stresses, Northern Corn Leaf Blight (NCLB) also known as Turcicum Leaf Blight (TLB), is the most important disease of maize caused by hemi-biotrophic pathogen *Setosphaeria turcica* anamorph *Exserohilum turcicum* formerly known as *Helminthosporium turcicum* [Pass] Leonard and Suggs". The disease has a widespread occurrence throughout the world and shows its presence in Asia, Africa, Europe and America. Low temperature, high humidity, heavy dew and high rainfall are conducive for the proliferation of the pathogen to cause the disease. *S. turcica* is differentiated into various races, some of the common races being 0, 1, 2, 3, 12, 23, 23 N, 123 N, identified based on their virulence against *Ht* (*Helminthosporium turcicum*) genes *Ht1, Ht2, Ht3, ht4, HtM, HtP, Htn1, HtNB, and rt* in maize plants^2^. *Ht* genes are known to confer qualitative resistance which is race specific, inherited by single genes, and mostly dominant in gene action, and were initially identified in different genetic backgrounds. Expression of *Ht* genes in maize plants and or avirulence genes of *S. turcica* are altered by environmental conditions like temperature and light intensity, creating unstable and less durable resistance. While most of the *Ht* genes are mapped on consensus genetic maps (www.maizegdb.org), some of them have been fine mapped and cloned. Chung et al.^3^ characterized and mapped a region on chromosome bin 8.06 from a maize hybrid DK888, and suggested that QTL NLB8.06_DK888_ was identical, allelic or closely linked and functionally related to *Ht2*. Hurni et al.^4^ cloned *Htn1* gene which confers quantitative and partial resistance to NCLB by delaying the onset of lesion formation. Using high resolution map based cloning, a receptor-like kinase gene was identified to be underlying the *Htn1* gene.

Qualitative resistance usually leads to a high level of resistance when avirulent races dominate the fungal population, whereas some *Ht* genes can easily turn ineffective in case of the occurrence of a virulent strain^2^. In temperate environments, where pathogen variability is less, pyramiding multiple *Ht* genes is a good strategy towards NCLB resistance breeding. In tropical environments with high pathogen abundance and variability, *Ht* genes were found to provide only partial resistance^5^. Broad-based quantitative resistance to NCLB is preferred in tropical environments, which could be achieved by quantitative disease resistance loci (dQTLs) alone, or in combination with effective *Ht* genes. dQTLs are loci of small effects and is less likely to be overcome by evolution of new pathogens, and therefore it is practically more useful to breeders^6^. Inheritance studies of quantitative NCLB resistance using classical methods have revealed predominantly additive gene action controlling the trait^7,8^.

Quantitative trait loci (QTL) or linkage mapping is an effective approach for studying complex and polygenic forms of disease resistance^9^. A number of mapping studies have been undertaken for identifying QTLs for NCLB resistance in varied germplasm and environments (Table 1). Previously reported QTL distribution for NCLB resistance has been very diffuse. Nevertheless, certain chromosomal regions are reportedly shared in multiple QTL mapping studies specifically on chromosomal bin 1.03–06, 4.04–06, 5.04–07, 8.02–03, 8.05–06 and 9.02–04 (Table 1). Meta-QTL studies on resistance to multiple foliar diseases identified about 147 multiple disease resistance mQTLs for three foliar diseases, NCLB, Southern leaf blight (SLB) and Gray leaf spot (GLS) and identified bins 3.04–08, 5.04–07, and 8.05–06 that are significant for resistance to these diseases^10^. QTLs on chromosome 3 bin 3.04–08 has been identified in many studies for NCLB and SLB^11^. QTLs on chromosome bin 5.04 and 5.06–07 were detected in different mapping studies for NCLB and GLS resistance^11^. QTLs on chromosome bin 8.05/8.06 has been detected in most of the QTL mapping studies for NCLB, where the major genes *Ht2* and *Htn1* are also mapped. This region has also been found to be important for resistance to other disease like GLS, common rust and common smut^12–15^. Meta-QTLs identified for multiple foliar diseases on chromosome 8 bin 8.08 were also found to be associated with two Nucleotide Binding Site (NBS) family of R genes. Meta-QTL analysis have also revealed that chromosome 8 possesses a cluster of QTLs and significant real (consensus) QTLs for NCLB, GLS and SLB with confidence interval (CI) lesser than 5 cM^10^ .

**Table 1:** Summary of selected genetic mapping studies for NCLB resistance using different mapping populations in various genetic backgrounds.

S. No	Chr	Bin	Markers	Lines	Mapping population	Trait	References
1	1, 2, 3, 4, 5, 8, 9 and 10	1.03, 1.05, 2.05, 4.05, 5.04, 8.03, 9.03	SNPs	NC304, NC344, Ki3, NC262, Oh7B, H100	8 BC3F4:5 population (1,611 lines)	AUDPC, LS means	^59^
2	2, 3, 4, 5, 8 and 9	2.05, 3.04, 4.05, 5.04, 8.03, 9.03 and 9.04	SNPs	NC304, NC344, Ki3, NC262 H100	12, F2:3 families	DLA	^11^
3	1, 4, 5, 6, 7, 8, 9 and 10	1.01, 4.04, 7.02, 8.03, 9.03 and 10.04	SNPs	Qi319, Ye478	314 RILs	Disease score and lesion size	^55^
4	4	4.01/4.05, 4.08/4.10	SSR	CM 212, CM 338	F2:3 families	PDI, AUDPC-PDI, LA and AUDPC-LA	^62^
5	1, 3, 5, 7 and 9	1.03, 3.08, 5.04, 7.05 and 9.03	SNPs	K22, BY815	207 RILs	DS	^52^
6	1, 2, 3, 4, 6, 8 and 9	1.06, 2.00–2.01, 2.02, 3.05, 3.09, 4.07–4.08, 4.08, 6.05, 6.07, 8.05. 8.07, 8.08, 9.02, 9.04	SSR	B73, Mo17	302, RILs	IP and WMD	^82^
7	1	1.02, 1.06	RFLP, SSR	Tx303, B73	82 TBBC3 introgression lines	AUDPC, IP, Lesion expansion, DLA, DS	^83^
8	8	8.06	SSR, SNPs	S11 9, DK888	17 F6 families	IP, primary DLA, DLA	^3^
9	1, 2, 6, 8	1.02, 1.05–1.06, 2.02–2.03, 6.05, 8.02, 8.05	SNPs and SSR	Ki14, B73	RILS	WMD	^58^
10	1, 2, 3, 4, 5, 8 and 9	_	RFLP	Lo951, CML202	194–256 F2:3 families	IP and AUDPC	^84^
11	1, 2, 3, 4, 5, 8 and 9	1.11, 2.03–04, 3.06, 4.08, 5.06–08, 8.05, 9.04–05	RFLP	Lo951, CML202	194–256 F2:3 families	DS and AUDPC	^53^
12	1, 2, 3, 4, 5, 6, 8 and 9	1.06–08, 2.06. 3.01, 3.03, 4.03, 4.06, 5.03, 5.04, 6.05–07, 8.02–03, 8.06, 9.02	RFLP and SSR	D32, D145	220 F3 families	DS	^85^
13	2, 3, 5, 7 and 8	_	RFLP	Mo17, B52	121 F2:3 families	DS, AUDPC	^50^
14	1S, 3L, 5S, 5L, 7L, 8L	_	RFLP	B52, Mol17	150 F2: 3 families	Lesion number, size of lesion and DS	^86^
15	1, 3, 5, 7 and 8	_	RFLP	Mol17, B52	150 F2: 3 families	DS	^87^

Molecular markers used in these studies were Single nucleotide polymorphism (SNPs), Simple sequence repeats (SSR), restricted fragment length polymorphism (RFLP), cleaved amplified polymorphic sites (CAPS). Phenotypic traits like area under disease progress curve (AUDPC), plant disease index (PDI), disease leaf area (DLA), incubation period (IP), disease severity (DS), weighted mean disease (WMD) were used for QTL analysis.

QTL mapping, though a powerful tool, has its own limitations such as i) limited number of recombination events during population development resulting in low mapping resolution, ii) only two alleles of each mapping population studied and iii) difficult to identify the positional candidate genes or to make strong inference on linkage relationships among other QTL identified^6^. In most of the QTL mapping studies, the mapping populations and breeding populations are unrelated, and hence the translation of the QTLs identified to breeding targets had been very few. GWAS, in assembled mapping panels representing the wide diversity in breeding programs, is another powerful tool to dissect complex traits and complements linkage mapping by improving mapping resolution. GWAS has been used to identify allelic variants that allow improved tolerance to various biotic and abiotic stresses in maize. Resistance to a large number of economically important and complex diseases of maize like Fusarium ear rot^16^, GLS^17,18^, head smut^19^, NCLB^6,20,21^, SLB^22^, sugarcane mosaic virus^23^, Maize streak virus^24^, Maize lethal necrosis^25^, sorghum downy mildew^26^ and tar spot^27^ have been dissected using GWAS. Several reports on GWAS for NCLB resistance in maize are available, mostly in temperate germplasm and environments. In a GWAS study conducted by Van Inghelandt et al.^21^, a large association mapping panel of 1487 inbred lines of temperate origin was used to dissect the genetic architecture of NCLB resistance, and reported association of significant SNPs on chromosomes 2, 5, 6 and 7 whereas, some of the SNPs were also identified on chromosomes 7 and 9 after correcting for flowering time variate. In a nested association mapping population of 4630 RILs, 208 SNPs associated with NCLB resistance on all 10 chromosomes of maize were identified, along with 29 QTLs, mostly with multiple loci^6^. Ding et al.^20^ studied the CIMMYT tropical maize germplasm that were phenotyped at different locations of Mexico and Africa for NCLB resistance and identified 12 SNPs on chromosome 3, 4, 6, 7, 8 and 10 for AUDPC, 14 SNPs on chromosome 1, 2, 3, 4, 6, 7, 9 and 10 for mean disease rating and 19 SNPs on chromosome 3, 4, 5, 7 and 10 for NCLB rating. In general, QTL mapping and GWAS studies in maize have revealed one important aspect that, NCLB resistance is a polygenic trait, and resistance due to major effects contributed by *Ht* genes was rare in most of the germplasm and environments studied. These studies suggested that there could be environment-specific and germplasm-specific moderate to large effect genomic regions controlling resistance to NCLB, which could be exploited in incorporating quantitative resistance to this important disease in breeding programs. Association mapping studies for NCLB resistance have been conducted by various research groups using temperate and tropical maize germplasm in American, African and European environments. However genome wide association studies using high density markers from large set of maize lines from Asian region are seldom reported. Therefore, the present research was designed to conduct GWAS for NCLB resistance under Asian conditions using tropical maize germplasm represented in three maize panels. These three panels represented maize germplasm from CIMMYT and several national partners, bred in different geographies across the tropics, and hence potentially selected in the presence of varied races of *S. turcica*. They represent most of the genetic diversity that is available across different tropical/sub-tropical geographies where CIMMYT breeding programs operate, and hence could be ideal resources for understanding the genetics of NCLB disease in South Asian tropics. In this study, apart from single SNP-based GWAS, we also identified haplotypes for resistance to NCLB within and across association panels representing differing germplasm backgrounds.

## Results

### Phenotypic evaluation for resistance to NCLB

**Figure 1:**
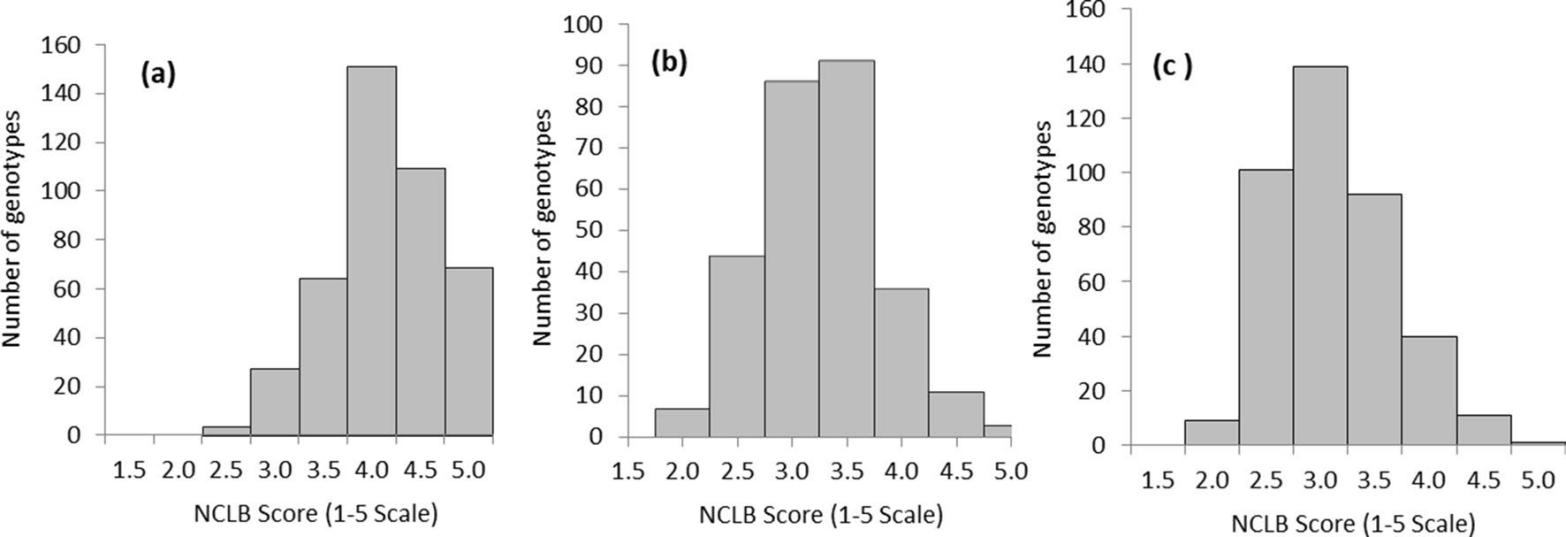
Phenotypic distribution of NCLB scores of (**a**) CAAM (**b**) DTMA and (**c**) IMAS panels on 1–5 scale with score 1 considered as highly resistant and score 5 as highly susceptible.

Subsets of three AM panels, CIMMYT Asia Association Mapping (CAAM), Drought Tolerant Maize for Africa (DTMA) and Improved Maize for African Soils (IMAS), consisting of 376, 224 and 324 lines, respectively were evaluated for NCLB resistance across different locations/years in India. Disease severity was high in the CAAM panel, with maximum score of 5.00 on a scale of 1.00–5.00 over all the three locations, with minimum disease score of 2.02, 1.50 and 1.46 at Mandya, Arabhavi and Kashmir, respectively. The average disease score across locations was 3.74. Broad-sense heritability (*h*^2^) was moderate to high (0.58–0.70) across individual locations with presence of significant genotypic variance (*P* value ≤ 0.001). DTMA panel at Mandya observed a mean of 2.85 with minimum disease score of 1.97 and maximum score of 4.76. Broad sense heritability estimated was 0.53, with highly significant genotypic variance (*P* value ≤ 0.001). Similarly, NCLB scores in IMAS panel ranged from 1.5–4.00 at Mandya in the first year and 2.00–5.00 in the second year, with mean rating of 2.55 and 3.44 during the two years, respectively. Overall analysis across the years revealed an average disease score of 3.00 with a maximum score of 4.55 and a minimum score of 1.96 where overall heritability (*h*^2^) estimate of 0.54 was observed with 0.48 in season 1 and 0.74 in season 2, respectively. IMAS panel also revealed significant genotypic variance (*P* value ≤ 0.001) (Table 2). The frequency distribution of mean NCLB disease ratings followed a near normal pattern in CAAM, DTMA and IMAS (Fig. 1). All the three AM panels revealed a significantly negative genotypic correlation between NCLB scores and days to anthesis (DA) (*P* value ≤ 0.001) (Table 3). Hence, best linear unpredicted estimates (BLUPs) were estimated using DA as a covariate to further conduct GWAS for NCLB resistance in all association panels.

**Table 2:** Single and across locations summary statistics, variance components and heritability estimates of the Northern corn leaf blight (NCLB) scores for CIMMYT Asia Association Mapping (CAAM), Drought Tolerant Maize for Africa (DTMA) and Improved Maize for African Soils (IMAS) panels.

Panels	Location/year	Mean	Min	Max	Genotypic variance	G × Location/year variance	Error variance	Heritability
CAAM	Mandya	3.899	2.020	5.000	0.178**	–	0.256	0.582
CAAM	Arabhavi	3.576	1.500	5.000	0.482**	–	0.410	0.701
CAAM	Kashmir	4.193	1.460	5.000	0.437**	–	0.475	0.648
	Across	3.744	2.120	5.000	0.112**	0.241**	0.326	0.454
DTMA	Mandya	2.851	1.970	4.760	0.147**	–	0.260	0.531
IMAS	Mandya-S1	2.555	1.500	4.000	0.120	–	0.130	0.479
IMAS	Mandya-S2	3.444	2.000	5.000	0.385	–	0.133	0.744
	Across	3.001	1.968	4.551	0.157**	0.099	0.134	0.541

** *P* value ≤ 0.001.

**Table 3:** Genetic correlation between the Northern corn leaf blight (NCLB) scores and days to anthesis (DA) for CIMMYT Asia Association Mapping (CAAM), Drought Tolerant Maize for Africa (DTMA) and Improved Maize for African Soils (IMAS) panels.

Panels	Trait	DA
CAAM	NCLB	− 0.4852**
DTMA	NCLB	− 0.1993**
IMAS	NCLB	− 0.3322**

*DA* days to anthesis.

**P value ≤ 0.001.

### Principal component analysis and linkage disequilibrium (LD) decay

**Figure 2:**
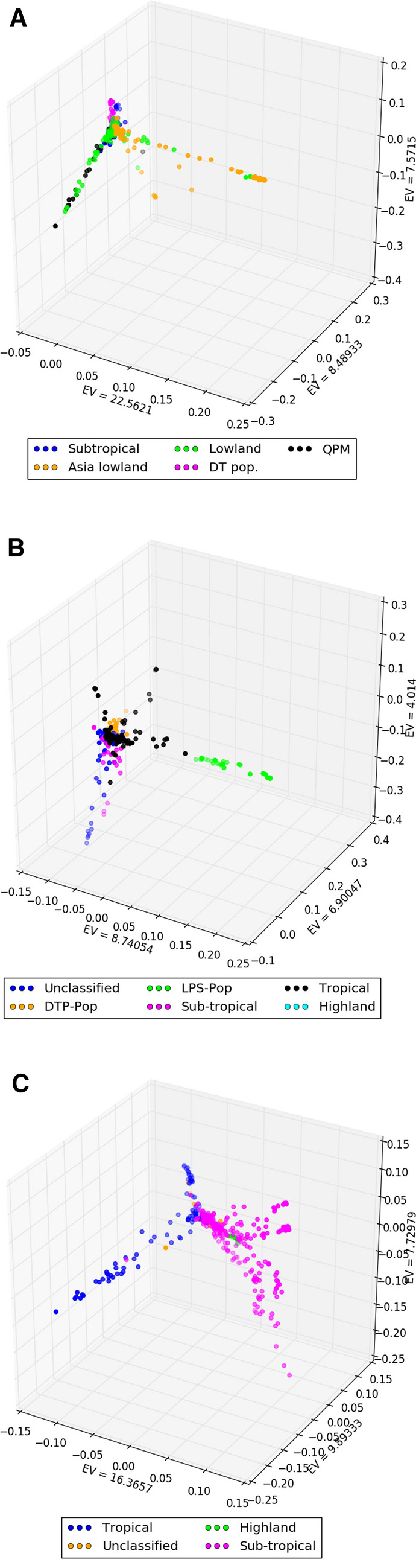
Population structure based on the first three Eigen values of principal components (PC) analysis of (**A**) CAAM panel using 64,344 SNPs (**B**) DTMA panel using 69,254 SNPs and (**C**) IMAS panel using 69,286 SNPs. Different coloured clusters represented the adaptation pattern of the three panels.

Principal Component Analysis (PCA) was performed by using the high density Genotyping by Sequencing (GBS) data, filtered for a call rate > 0.9, minor allele frequency > 0.1 and LD pruning at *r*^2^ = 0.5. The first three principal components of each panel are depicted in Fig. 2. The CAAM panel showed moderate structure, in which the Asian lowland lines partially separated from the CIMMYT lowland germplasm whereas, QPM lines grouped with the CIMMYT lowland germplasm. The DTMA panel did not exhibit substantial differential clustering of the various lines of different adaptation categories, other than the clear separation of La Posta Sequia (LPS) lines developed under CIMMYT’s LPS population improvement program, mainly for drought tolerance. IMAS panel also revealed moderate structure with clear separation of tropical and sub-tropical maize lines, with overlapping highland and sub-tropical lines. The first three PCs explained 38.56, 19.65 and 33.98 per cent variance in CAAM, DTMA and IMAS panel, respectively.

**Figure 3:**
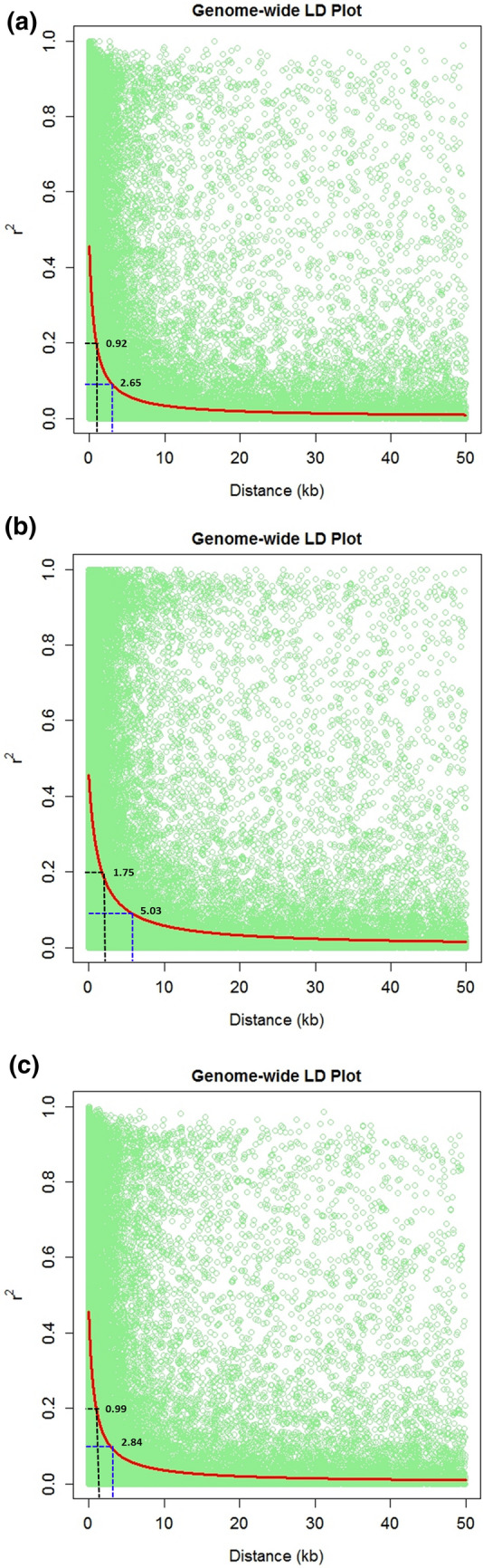
Linkage disequilibrium (LD) plot illustrating the average genome wide LD decay of (**a**) CAAM (**b**) DTMA and (**c**) IMAS panel using the SNPs with call rate 0.9 and minor allele frequency 0.1. The values on the Y-axis represents the squared correlation coefficient *r*^2^ and the X-axis represents the genetic distance in kilo bases (Kb).

The genome wide linkage disequilibrium (LD) was plotted as LD (*r*^2^) between adjacent pairs of markers versus the distance between adjacent markers in Kb (Fig. 3). Genome wide LD plot displayed the LD-decay in CAAM panel as 2.65 Kb at *r*^2^ = 0.1 and 0.92 Kb at *r*^2^ = 0.2, with chromosome 7 showing the fastest LD-decay (1.79 Kb at *r*^2^ = 0.1 and 0.62 Kb at *r*^2^ = 0.2).Chromosome 8 displayed the slowest LD-decay (4.69 Kb at *r*^2^ = 0.1 and 1.63 Kb at *r*^2^ = 0.2). DTMA panel showed the slowest genome wide LD-decay amongst the three panels with LD decay of 5.03 Kb at *r*^2^ = 0.1 and 1.75 Kb at *r*^2^ = 0.2. Chromosome wise LD-decay revealed that the fastest decay was in chromosome 6 (3.22 Kb at *r*^2^ = 0.1 and 1.11 Kb at *r*^2^ = 0.2) while chromosome 8 showed the slowest decay (12.81 Kb at *r*^2^ = 0.1 and 4.43 Kb at *r*^2^ = 0.2) decay. IMAS panel showed the genome LD-decay of 2.84 Kb at *r*^2^ = 0.1 and 0.99 Kb at *r*^2^ = 0.2 with chromosome 6 (2.02 Kb at *r*^2^ = 0.1 and 0.70 Kb at *r*^2^ = 0.2) and chromosome 8 (5.24 Kb at *r*^2^ = 0.1 and 1.83 Kb at *r*^2^ = 0.2) showed the fastest and slowest LD-decay, respectively (Supplementary Table 1).

### GWAS for NCLB resistance

**Figure 4:**
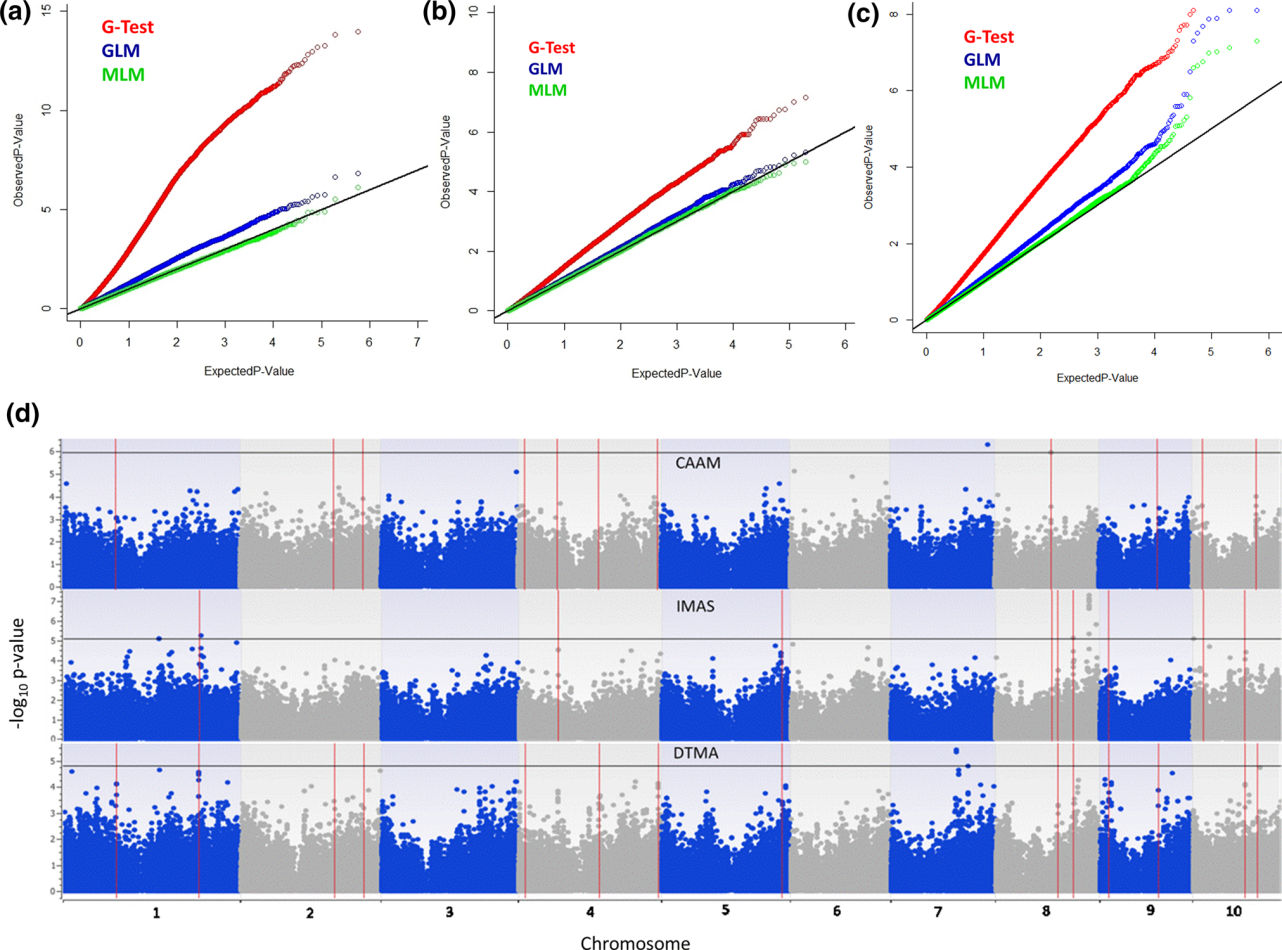
Inflation depicted by Q–Q plots of observed versus expected -log_10_ (*P* values) plots for NCLB using the naïve association model (G-test), GLM (G + Q) and MLM (G + Q + K); G = genotype (fixed), Q = ten principal components (fixed), K = kinship matrix (random) for (**a**) CAAM panel (**b**) DTMA panel and (**c**) IMAS panel; Highly significant SNPs identified from MLM model using Manhattan plot (**d**), plotted with the individual SNPs on the X-axis and − log_10_ *P* value of each SNP on the Y-axis for the three panels, CAAM, IMAS and DTMA. The horizontal line shows the cut off *P* value and the vertical lines represent the common haplotypes identified in haplotype regression analysis across different panels for NCLB resistance.

A robust subset of SNPs from high density imputed 955 K GBS genotypic data was used to conduct GWAS with 293,606, 297,437, and 309,608 SNPs after following the filtration criteria of call rate ≥ 0.7 and minor allele frequency ≥ 0.05 in CAAM, DTMA and IMAS panels, respectively. Naïve or G-test association model showed highest genomic inflation; whereas MLM model corrected for both population structure and kinship revealed the least genomic inflation as observed in the Quantile–Quantile (QQ) plots (Fig. 4). Therefore highly significant associations for NCLB resistance in the panels were ascertained based on MLM analysis. The narrow sense heritability for NCLB resistance in CAAM, DTMA and IMAS panels was 0.56, 0.52 and 0.53, respectively based on the IBS kinship matrix employing all SNPs used in GWAS. A total of five SNPs were identified to be associated with NCLB resistance in CAAM panel with *P* values ranging from 5.27 × 10^–07^ to 8.22 × 10^–06^. Three SNPs in DTMA panel and 14 SNPs in IMAS panel were identified in MLM analysis, with *P* values ranging from 3.76 × 10^–06^ to 1.63 × 10^–05^ and 4.98 × 10^–08^ to 8.60 × 10^–06^ in DTMA and IMAS panels, respectively (Table 4). SNP S7_165196774 located on chromosome 7 and three other SNPs S8_95422954, S8_95422964 and S8_95422973 with closely placed physical co-ordinates on chromosome 8 showed the lowest *P* values for NCLB resistance in the CAAM panel explained phenotypic variance ranging from 5.32 to 6.68%. Similarly in DTMA panel, GWAS identified three highly significant SNPs on chromosome 7 (S7_110282525, S7_110282502 and S7_131034143), explaining phenotypic variance ranging from 8.45 to 9.65%. A group of 8 SNPs located at close physical co-ordinates near 157 Mb on chromosome 8 were among the 14 SNPs which were identified to be showing most significant association with NCLB in the IMAS panel (Table 4). SNP S8_157987611 was observed to be significantly associated at a *P* value of 4.98 × 10^–08^ and explained 9.10% of phenotypic variance. GWAS conducted on different panels identified several SNPs that have co-localized physical co-ordinates within chromosomal bins where *Ht* genes or QTLs for NCLB resistance were previously reported. Predicted gene annotations in the B73 maize reference genome version 2 (http://ensembl.gramene.org/Zea_mays) were studied to identify the genes based on the SNPs associated with NCLB resistance. Several significant SNP associations in these three GWAS studies were located within genes with functional domains leading to biotic and abiotic stress tolerance, immune response, metabolism, plant development and maturity and responses to abiotic stresses (Table 4).

**Table 4:** Highly significant Single nucleotide polymorphisms (SNPs) identified in GWAS analysis of CAAM, DTMA and IMAS association panels that were evaluated NCLB resistance.

Association panels	SNPs	Chr	*P* Value	Favourable allele	R^2^ (%)	Co-localized gene annotation	Functional domain	Crops reported	Function	References
CAAM	S7_165196774	7	5.27 × 10^–07^	A	6.688	GRMZM2G116426	Alpha/beta-Hydrolases superfamily protein	Plants	Plant immune response	^63^
	S8_95422954	8	1.24 × 10^–06^	A	6.263	GRMZM2G337616	Cytosolic endo-beta-N-acetylglucosaminidase 1	Arabidopsis, Rice, Maize, Tomato	Degradation of glycol proteins, Plant development and fruit maturation	^88,89^
	S8_95422964	8	1.24 × 10–^06^	T	6.263					
	S8_95422973	8	1.24 × 10^–06^	G	6.263					
	S6_7150106	6	8.22 × 10^–06^	C	5.324	GRMZM2G057091	Chloroplast J-like domain 1	Arabidopsis	Abiotic stresses	^90^
DTMA	S7_110282525	7	3.76 × 10^–06^	G	9.659	GRMZM2G334165	Protein kinase superfamily protein	Plants	Metabolism, cell division, defence	^91^
	S7_110282502	7	4.87 × 10^–06^	T	9.447					
	S7_131034143	7	1.63 × 10^–05^	G	8.45	GRMZM2G031613	Subtilisin-like protease SBT5.3	Plants, Arabidopsis	Plants defence mechanism and crop improvement	^92^
IMAS	S8_157987611	8	4.98 × 10^–08^	C	9.102	GRMZM2G319130	Putative regulator of chromosome condensation (RCC1) family protein	Arabidopsis, Soybean	Disease resistance against necrotrophic pathogens F *Phytophthora sojae*	^66,67^
	S8_157986156	8	7.67 × 10^–08^	T	8.857					
	S8_157987595	8	9.49 × 10^–08^	C	8.736					
	S8_157985530	8	1.06 × 10^–07^	T	8.675					
	S8_157987565	8	1.74 × 10^–07^	C	8.392					
	S8_157986163	8	2.20 × 10^–07^	G	8.258					
	S8_157987702	8	2.56 × 10^–07^	A	8.171					
	S8_170782575	8	1.53 × 10^–06^	A	7.152	GRMZM2G130831	Cation/H( +) antiporter 15	Grapevine	Salt tolerance	^93^
	S8_157987471	8	4.86 × 10^–06^	C	6.487	GRMZM2G319130	Putative regulator of chromosome condensation (RCC1) family protein	Arabidopsis, Soybean	Disease resistance against necrotrophic pathogens F Phytophthora sojae growth and development	^66,67^
	S1_233446021	1	5.98 × 10^–06^	C	6.368	GRMZM2G001764	Protein NRT1/ PTR FAMILY 5.2	Arabidopsis		^94^
	S8_131534569	8	7.95 × 10^–06^	G	6.204	GRMZM2G351921	–	–	–	–
	S1_161617710	1	8.28 × 10^–06^	C	6.18	GRMZM2G060690	Cyclin-A2	Alfalfa	Meristem formation cell division and cell morphogenesis	^95^
	S1_161617735	1	8.28 × 10^–06^	C	6.18					
	S10_2032185	10	8.60 × 10^–06^	G	6.159	GRMZM2G180150	brick3	Maize		^96^

### Haplotype detection and regression analysis for the trait

A set of 842 SNPs in the bottom 0.1 percentile of the distribution in each GWAS study detected 112 haplotype blocks across the 10 chromosomes. Haplotype Regression Analysis (HTR) was carried out with 112 haplotypes on NCLB BLUP estimates of individual maize panels separately. HTR analysis in the CAAM panel identified 21 haplotype blocks with FDR value ≤ 0.05 that explained 1.98–8.46% variance (Supplementary Table 2). Thirty nine haplotypes were found to be significantly associated with NCLB disease rating in DTMA panel with explained phenotypic variance of 2.64–13.90% (Supplementary Table 3). In IMAS panel, 38 haplotype blocks were detected to be associated with NCLB resistance explaining phenotypic variance ranging from 1.71 to 11.50% (Supplementary Table 4). HTR analysis identified 17 common haplotypes having a significant effect (FDR ≤ 0.05) on the trait in at least two different AM panels spread across seven chromosomes (1, 2, 4, 5, 8, 9 and 10), each consisting of 2–10 SNPs (Table 5; Fig. 4). The proportion of variance explained by these common haplotype blocks ranged from 1.71 to 9.42%. No haplotype was identified to have a significant effect on the trait in all three AM panels. CAAM and DTMA panels shared eight common haplotypes with significant effect on the trait, whereas six and three common haplotypes were identified between DTMA and IMAS panels and CAAM and IMAS panels, respectively that are significantly associated with NCLB disease.

**Table 5:** Common haplotypes identified across panels for resistance to NCLB in haplotype regression analyses of CAAM, DTMA and IMAS panels.

Haplotype block	Chromosome	Markers used	*P* value	R^2^ (%)	FDR value	Favourable alleles	Panel
Hap_1.1	1	S1_90892964, S1_90892966, S1_90892967, S1_90892970	0.00355	2.789	0.020925	TTTG	CAAM
			2.43 × 10^–05^	8.711	0.000247	TTTG	DTMA
Hap_1.2	1	S1_230882239, S1_230882260, S1_230882354, S1_230882360, S1_230882374	0.001093	5.334	0.00583	CTATG	DTMA
			0.001229	3.544	0.004589	GCGAC	IMAS
Hap_2.1	2	S2_158673333, S2_158673408, S2_158673422, S2_158673429, S2_158674708	0.005127	2.198	0.028709	CGCGT	CAAM
			0.004642	3.585	0.01677	CGTGA	DTMA
Hap_2.2	2	S2_208538471, S2_211029206	0.001659	2.889	0.012385	TA	CAAM
			0.013012	2.809	0.039387	TA	DTMA
Hap_4.1	4	S4_11081178, S4_11081212	1.15 × 10^–05^	6.792	0.000259	AA	CAAM
			0.00407	4.893	0.015719	AA	DTMA
Hap_4.2	4	S4_66558784, S4_66558850, S4_66558853, S4_66558873	0.001331	2.904	0.01065	GAGC	CAAM
			7.97 × 10^–07^	7.277	1.79E−05	ATAT	IMAS
Hap_4.3	4	S4_136671077, S4_136671078, S4_136671079, S4_136671091	0.002837	2.921	0.017651	GCTT	CAAM
			3.67 × 10^–05^	8.470	0.000342	GCTT	DTMA
Hap_4.4	4	S4_236938491, S4_236938493, S4_236938494	0.002117	2.608	0.013944	TTC	CAAM
			0.000433	5.687	0.002695	CCT	DTMA
Hap_5	5	S5_204091738, S5_204091741	0.002912	3.993	0.013044	GA	DTMA
			1.40 × 10^–08^	9.123	7.82E−07	GA	IMAS
Hap_8.1	8	S8_95422954, S8_95422964, S8_95422973	2.89 × 10^–05^	6.445	0.000405	CAT	CAAM
			0.011805	2.342	0.037776	CAT	IMAS
Hap_8.2	8	S8_105321803, S8_105321804, S8_105321807, S8_105321808, S8_105321834	0.001959	4.391	0.009542	AGCAA	DTMA
			0.000802	3.445	0.003096	TAAGG	IMAS
Hap_8.3	8	S8_131534491, S8_131534569	0.000479	5.429	0.002824	CA	DTMA
			0.015592	1.713	0.047197	CA	IMAS
Hap_9.1	9	S9_14906566, S9_14906568, S9_14906569, S9_14906572, S9_14906573, S9_14906574, S9_14906575, S9_14906576, S9_14906577, S9_14906578	0.015371	3.778	0.045304	AATGATTTTT	DTMA
			0.00023	4.892	0.001121	AATGATTTTT	IMAS
Hap_9.2	9	S9_99293028, S9_99293080	0.000392	4.476	0.003991	GA	CAAM
			0.000277	6.473	0.001828	AC	DTMA
Hap_10.1	10	S10_18140579, S10_18140580, S10_18140584, S10_18140587	0.001221	3.500	0.010521	GCCC	CAAM
			0.010931	2.307	0.036007	GCCC	IMAS
Hap_10.2	10	S10_88268774, S10_88268837	5.51 × 10^–06^	9.430	7.72E−05	GG	DTMA
			0.009943	2.009	0.033747	GG	IMAS
Hap_10.3	10	S10_109345864, S10_109345865, S10_109345872	1.47 × 10^–05^	5.090	0.000274	ATA	CAAM
			0.012477	2.992	0.038816	CCT	DTMA

## Discussion

NCLB is an important foliar disease of maize in almost all temperate and tropical maize growing regions of the world. Resistance for NCLB in maize can be achieved through breeding using qualitative and quantitative resistance, either separately or together. However, resistance provided by qualitative/major genes becomes ineffective in the presence of virulent strains. Tropical environments show high pathogen abundance and high genetic diversity which leads to inflated disease severity, and hence the chances of breakdown of resistance are high. Compared to other grass crops like rice and wheat, majority of disease resistance deployed by maize breeders are quantitative in nature, and not qualitative^28^. It was also noted that the major genes influencing NCLB resistance have high environmental dependence with regard to light and temperature^29^, and act like partial/quantitative resistance in some environments. Resistance to NCLB is considered to be a mandatory trait in breeding successful maize varieties across the tropics, and hence is an important breeding target. Therefore identifying, validating and deploying high value genomic regions for the trait will help in achieving enhanced genetic gains for the trait. Targeted molecular breeding for traits demand genetic mapping and molecular characterization of the functional genomic regions associated with the trait^30^. Association mapping utilizes the ancestral recombination events in a natural population to make marker-phenotype relations^31^. It has several advantages over linkage mapping such as, (1) existing population can be used rather than developing new bi- parental population for mapping. (2) Large number of alleles can be surveyed (3) Higher mapping resolution and (4) Lesser research time^30,32^.

The three association mapping panels used in this study represent most of the genetic diversity that is available across different geographies where CIMMYT breeding programs operate, and hence could be ideal resources for understanding the genetics of NCLB disease in Asia. The Trial means for NCLB scores of IMAS and DTMA panels were lesser compared to CAAM panel at Mandya, which is a location with high disease severity and where all the three panels were evaluated, indicating that higher levels of resistance is available in the African and Latin American CIMMYT germplasm, as compared to CIMMYT-Asia germplasm. One of the reasons could be that the DTMA and IMAS panel included lines predominantly adapted to Sub Saharan Africa (SSA), where large number of lines were evaluated for foliar diseases like GLS, NCLB and common rust by collaborators through a regional maize disease nursery project (REGNUR)^33^. However the CAAM panel lines were bred or/and selected for the Asian environments, and had a history of breeding for resistance to diseases like downy mildews^34,35^. The CAAM panel evaluated at three locations observed highest disease score mean at Kashmir, located at higher altitude in the northern boundary of India, which may be due to highly congenial environment for disease development owing to cool and humid weather, and probable presence of more virulent races of the pathogen at that location. For the phenotyping trials, artificial inoculation was conducted at all locations with the pathogen sources collected from respective locations.

For principal component and kinship analysis, SNPs fulfilling the criteria of CR ≥ 0.9, MAF ≥ 0.1 and LD pruned at an *r*^2^ threshold of 0.5 were used. LD-pruning was done to reduce the confounding effects due to large blocks of SNPs that have strong LD with each other^36^. There was only moderate structure observed in the three panels, with no clear differentiation of major adaptation groups, except in the IMAS panel. The CIMMYT maize germplasm was not found to have strong population structure in various earlier studies^25,26,37^. George et al.^38^ observed that CIMMYT’s tropical and sub-tropical lines in the Asian region possess significant genetic diversity that did not allow a clear distinction into separate clusters. Warburton et al*.*^39^ observed that the CIMMYT pools and populations which served as the germplasm sources for derivation of many breeding lines in the tropical and sub-tropical adaptation groups had a large amount of diversity within, than between source populations. This heterogeneous nature of the CIMMYT populations was suggested to be responsible for the lack of a well-defined population structure in the germplasm. A rapid LD decay was observed in all the panels (0.9 kb at *r*^2^ = 0.2 for CAAM, 1.75 kb at *r*^2^ = 0.2 for DTMA and 0.99 kb at *r*^2^ = 0.2 for IMAS panel). Lu et al*.*^40^ found that the LD decay distance in temperate maize germplasm (10–100 kb) was 2 to 10 times higher than that of tropical maize germplasm (5–10 kb). Our results were more similar to the finding by Romay et al.^41^ that LD decays much more rapidly in the tropical germplasm to about 1 kb at *r*^2^ = 0.2. The higher LD decay in tropical germplasm suggests the more diverse genetic base that resulted from the historic recombination events and might have more rare alleles than temperate germplasm^42^. The LD decay was different for the 10 chromosomes in all panels, with the slowest decay observed in chromosome 8 consistently in all the panels studied. This was also observed by Suwarno et al.^43^ in a Carotenoid association mapping panel comprising of tropical, sub-tropical and a small proportion of temperate lines. Pace et al.^44^ have also observed this pattern in a sub-set of AMES panel, which is predominantly a temperate maize lines panel. This is an interesting observation and will require further probing to understand the reasons behind the slower LD decay in chromosome 8 and its implications in molecular breeding applicability in terms of traits like resistance to NCLB, with genomic regions conferred by genes/QTL located on Chromosome 8.

The single locus mixed linear model was used after correcting for population structure and familial relationships (kinship), for conducting GWAS in all the panels to reduce the genomic inflation. Highly significant SNPs associated with NCLB resistance were selected based on the significance threshold corrected for multiple testing corrections, taking average extent of genome-wide LD into consideration^45^. A Total of 22 SNPs significantly associated with NCLB resistance were identified on chromosomes 1, 6, 7, 8 and 10. The most significant association in the CAAM panel was with SNP S7_165196774 (*P* value 5.27 × 10^–7^), at 165.19 Mb, in the bin 7.04 (www.maizegdb.org, Maize B73 RefGen_V2), co-located within the physical interval of markers flanking the major gene *Ht3*^21^. *Ht3* has been introgressed from *Tripsacum floridanum* into maize^46^and *Ht3* gene provides resistance against the *S. turcica* races 0, 1, 2, N, 12 and 2N^47^, by inhibiting the extension of chlorotic spots and decreasing the production of spores by the pathogen^46^. *S. turcica* races 0, 1 and 2 are prominent in the Asian countries like China and India^48^, and *Ht3* could be effective against these races. Considering the most significant SNP identified could possibly be in LD with *Ht3*, owing to its physical location, it provides a strong lead to follow up for future NCLB resistance mapping and deployment efforts. A glutathione S. transferase (*GST)* gene, belonging to a plant-specific clade implicated in defence has also been identified in bin 7.04 that confers multiple disease resistance to NCLB, SLB and GLS^49^. In the DTMA panel, two highly significant SNPs (S7_110282525, S7_110282502), closely located at 110 Mb (bin 7.02) on chromosome 7, were found to be associated with NCLB resistance. Van Inghelandt et al*.*^21^ identified SNPs on bin 7.02 associated with NCLB resistance in a GWAS of 1487 maize inbred lines representing elite European and North American germplasm. Similarly, another significantly associated SNP identified in the DTMA panel (S7_131034143) was located on chromosomal bin 7.03. Dingerdissen et al*.*^50^ identified a QTL in this chromosomal bin for area under disease progression curve (AUDPC) trait in F_2:3_ lines derived from Mo17 and B52 at Embu, Kitale and Muguga, Kenya against the races 0 and N prevalent in Kenya.

In the IMAS panel, eight closely located SNPs, located at 157 Mb on bin 8.06 of chromosome 8, were identified to be the most significant association to NCLB resistance. In the maize genome, chromosome 8 (bin 8.05–8.06) is known to harbour genes for various defence pathways, and could be considered as one of the “complex, important and interesting” genomic region in terms of maize disease resistance, and NCLB resistance in particular^3^. It is considered as an important genomic region for many dQTLs and major genes like *Ht2* and *Htn1* for NCLB resistance^28^. Though there are apparent differences in the definition of qualitative and quantitative resistance, sometimes, pure qualitative and quantitative resistance are considered to be two ends of the same continuum and most resistance genes exist between the two extremes^51^. Chung et al.^3^ fine mapped a major QTL explaining a large proportion (14–62%) of phenotypic variance in NCLB resistance for the race 0 and 1 on bin 8.06, and it was described as either identical or allelic or closely linked and functionally similar to the major gene *Ht2*, which is partially dominant and is effective against the 0, 1, 3 and N races. *Htn1* gene is also present in this genomic region, which is known to delay the lesion development up to four weeks after infection, reduce the number of lesion and delay the sporulation and found to be effective against most NCLB races^5^. *Htn1* was cloned and found to be a wall associated receptor-like protein, and confer quantitative and partial resistance against NCLB^4^. Many other studies have also identified NCLB QTLs in these chromosomal bins. Poland et al.^6^ identified a large effect QTL at 152.2 Mb on bin 8.06, segregating in multiple NAM families. Similarly Chen et al.^52^ also identified a QTL in bin 8.06 for lesion width, while studying a RIL population. A major QTL was identified for AUDPC on chromosome 8 between the bins 8.05–8.06 in F_2:3_ populations studied for NCLB resistance^53^. Recently, a study conducted on a nested near isogenic line library for resistance to NCLB, also identified NILs with introgressions across centromeric region of chromosome 8 (bin 8.05), which overlaps two major genes *ht2* and *htn*^54^. The fact that one of our mapping panels also identified a strongly associated set of closely located SNPs in this important chromosomal bin, indicated possible presence of a quantitatively expressed major gene or dQTL in this region present in the genetic background of the maize lines of this particular panel. *S. turcica* races prevalent in the locations that have been used for phenotyping in the present study are not yet reported, but physiological race 1 of *S. turcica* of maize was reported in the adjoining areas^48^. Wang et al*.*^55^ identified two minor QTLs on bin 8.03 associated with NCLB resistance in a RIL population. Our study also identified a group of closely linked three SNPs on bin 8.03 of chromosome 8 (S8_95422954, S8_95422964, S8_95422973) associated with NCLB resistance in the CAAM panel.

Haplotype regression analysis identified 17 haplotype blocks that are common across at least two panels among the three panels studied, and hence considered to be candidates for further studies towards NCLB resistance in Asian tropics. The use of haplotypes increase the phenotypic variance explained, and thus allows the identification of genomic regions responsible for controlling a large part of variation in the trait of interest^56^. The size of the haplotype block depends on the degree of LD present in the population^57^. Haplotype information can be beneficial when identifying marker phenotype associations and can offer advantages for the genetic dissection of loci underlying the complex trait^20^. Out of the 17 common haplotypes identified to be significant for NCLB resistance across different AM panels, eight haplotypes were shared between CAAM and DTMA panel, six were common between DTMA and IMAS panels and three haplotypes were shared between CAAM and IMAS panels. Haplotype Hap_1.1 was identified on chromosomal bin 1.06 in the CAAM and DTMA panels, and this bin is considered to be an important genomic region controlling resistance to multiple foliar diseases like NCLB, Stewart’s wilt, GLS and SLB^58–60^. Jamann et al.^60^ identified a receptor-like kinase gene, *pan1*, that underlie a QTL for NCLB in this region. Similarly, the physical co-ordinates of the SNPs forming the haplotype block Hap_9.1 identified in DTMA and IMAS panels fall within the confidence interval of qMdr_9.02_ reported for multiple disease resistance to NCLB, GLS and SLB^61^. Another haplotype identified on chromosomal bin 9.03 (Hap_9.2) identified in CAAM and DTMA panels was found to be located in close physical proximity to two closely spaced SNPs at 99.41 Mb identified in the Iodent material in a GWAS study conducted by Van Inghelandt et al*.*^21^. QTLs for resistance to NCLB and for multiple disease resistance on chromosomal region 4.05 have been identified in various studies^11,59,62^, and our study also identified two haplotype blocks Hap_4.2 and Hap_4.3 in this chromosomal bin. Overall, it was found that several SNPs/haplotypes identified in this study are in close proximity to previously reported major genes and QTL clusters, but many novel genomic regions were also discovered that could be environment and germplasm-specific.

Some of the SNPs identified in this study were found to be located in annotated genes (B73 RefGen_V2) with functional domains implicated in defence mechanisms in crops like maize, rice, and Arabidopsis. Highly significant SNP S7_165196774, identified in the CAAM panel is located in the gene GRMZM2G116426, having functional domains of alpha/beta-Hydrolases (ABH) superfamily proteins. ABHs support a variety of unique catalytic functions for defence and hormone regulation^63^. ABH esterase regulates the response of salicylic acid in plants, which is a key hormone to plant immune responses^64^. Highly significant SNPs identified in the DTMA panel on chromosome 7 are located within GRMZM2G334165 gene coding for protein kinase superfamily. Protein kinases play a central role in signalling during pathogen recognition and the subsequent activation of plant defence mechanisms. The microbial (pathogen) elicitors, also known as pathogen-associated molecular patterns (PAMPs), are recognized by the membrane-localized pattern recognition receptors (PRRs) of plants^65^. Transmembrane receptor kinases are one of the PRRs which help in plant defence mechanism. Eight significantly associated SNPs on chromosome 8 in the IMAS panel were found to be located in GRMZM2G319130 gene putatively coding for regulator of chromosome condensation (RCC1) family protein. RCC1 proteins contain plant specific disease resistance, zinc finger, chromosome condensation (DZC) domain^66^, and *RML3* gene implicated in resistance to *Leptosphaeria mculans* in Arabadopsis was found to have RCC1 domain. It was also found to be effective for broad spectrum resistance against several necrotrophic fungi*.* Two genes BQ081031 and BQ080005 encoding candidate regulators of RCC1 family protein were found to be down-regulated specifically in the resistant reaction following *Phytophthera*. *sojae* infection which causes stem and root rot in soybean^67^.

## Conclusion

From three GWAS panels genotyped at high density, and phenotyped for NCLB disease under artificial disease pressure in multiple environments in India, 22 significant SNP associations were identified. Seventeen haplotypes were identified which were significantly associated with the trait across two or more panels studied. Several SNPs/haplotypes identified in this study were located within or in close proximity to major genes like *Ht3*, *Ht2* and *Htn1* and many previously reported dQTLs, and multiple foliar disease resistant QTL. These regions will be candidates for further validation studies and possible utilization in the breeding programs in Asia. Considerable differences were observed among different germplasm in terms of resistance to NCLB, and hence it is suggested to bring together diverse sources of resistance alleles to improve resistance to NCLB.

## Materials and methods

### Plant material

Three association mapping panels CAAM, DTMA and IMAS panels assembled by CIMMYT, Global Maize Program were used to study genome wide association for NCLB resistance. The CAAM panel included 419 tropical/ sub-tropical lines from the different breeding programs of CIMMYT adapted to Asian ecologies. This diverse panel included the lines derived from the different source populations for drought, waterlogging, heat stress, acid soil tolerance and downy mildew resistant lines. The panel has early, medium and late maturing lines with predominantly yellow kernel color. This panel has been earlier studied for GWAS for traits like root traits under drought^68^ and resistance for sorghum downy mildew^26^. The DTMA panel consisted of 285 elite inbred lines which include CIMMYT’s drought tolerant (DT) lines, with reasonable resistance to foliar diseases and insect pests. Apart from drought tolerant lines derived from various selection cycles of the DT populations like DTP1, DTP2 and La Posta Sequia, the panel also included the elite set of lines from CIMMYT breeding programs in Latin America, eastern and southern Africa, and a large set of lines from the multiple borer resistant populations developed at CIMMYT, Mexico. The lines belong to medium to late maturity group with mostly white kernel color. GWAS was previously conducted in the DTMA panel for resistance to various biotic stresses like NCLB, Maize streak virus, Maize lethal necrosis, and Tar spot^20,24,25,27^. The IMAS panel constituted of 380 inbred lines which included elite CIMMYT Maize Lines (CMLs), lines developed from CIMMYT breeding programs in Kenya, Zimbabwe and Mexico, and lines developed by national partners in Kenya (KALRO) and South Africa (ARC). IMAS panel was earlier used in GWAS analysis of resistance to MLN^25^.

### Phenotypic evaluation

#### Screening sites

CAAM panel of 419 inbred lines were evaluated for NCLB at three high disease prevalence locations for one season at Mandya (12°N; 76°E; 695 masl; 705 mm/year average annual rainfall) Arabhavi (16.2213° N, 74.8229° E; 574 masl; 495 mm/year average annual rainfall) and Khudwani, Kashmir (33.5335° N, 74.9209° E, 1560 m asml, 680 mm/year annual rainfall). The sub-set of 285 lines of DTMA panel were evaluated for one season at Mandya and the set of 380 lines of IMAS panel were evaluated at Mandya for two seasons. CAAM and DTMA panels were evaluated as replicated trials with two replications using alpha lattice design. The IMAS panel was evaluated using complete block augmented design in 40 blocks, with block size 12 and the two checks (resistant check-CML451 and susceptible check- CML474), that were replicated in each block. These trials were conducted during the rainy season as the conditions were more congenial for disease development. All entries were planted in 2 m row plot using a spacing of 0.75 m between rows and 0.20 m between plants in each row.

#### Artificial inoculation

*S. turcica* strains were isolated from previous year’s diseased maize leaves. Infected leaves were cut into 5–10 mm small pieces, washed with 0.6% sodium hypochlorite for 1 min and rinsed with sterile distilled water for 3–4 times under aseptic conditions. Excess water was blot dried on sterile tissue paper and infected leaf pieces were placed on Petri plates carrying pure culture Potato Dextrose Agar (PDA). The plates werse incubated at 28 °C for 3–5 days, the growing hyphal tips were transferred to PDA allowed to grow for 8–10 days at 28 °C, conidia were isolated using single spore isolation method. Pure culture of *S. turcica* were maintained on PDA for further use.

For artificial inoculation in the field experiments, mass multiplication of fungal culture was done on sterile sorghum grains. Approximately 200–250 g of sorghum grains were autoclaved in 500 ml conical flask, and on attaining the normal room temperature, the grains were inoculated with pure culture of *S. turcica* earlier grown on PDA*.* Flasks were incubated at 28 °C for 15–20 days until the grains were uniformly covered with fungal growth. The cultured grains were dried and ground into powder and stored in paper bags until use. Trials were inoculated by putting 1 g of ground sorghum powder into the whorl of 30 days old maize crop and the process repeated at 40 days to avoid any escapes. Soon after the inoculations, plain water was sprinkled by manual sprayer of 15 L capacity on all fungus inoculated plants. This increased the humidity and leaf wetness necessary for disease development, and thus better and more reliable phenotyping data.

#### Disease scoring

NCLB symptoms started developing after a week of artificial inoculation, however symptoms became distinguishable after reproductive growth of the plants. Disease rating in trials was recorded two times, first score was taken at 65–70 days of crop, and the second or final scoring was taken on 75th–80th day. NCLB rating was recorded using 1–5 scale^69^; Score 1 = highly resistant (HR) where no infection or slight infection with few lesions scattered on lower two leaves 2 = resistant (R), Light infection with moderate number of lesions scattered on lower four leaves, 3 = moderately resistant (MR) moderate to heavy infection abundant number of lesions scattered on lower leaves and few lesions on the middle leaves below the cob 4 = Susceptible (S) heavy infection, abundant number of lesions scattered on lower and middle leaves and lesions spread up to the flag leaf and 5 = highly susceptible (HS) very heavy infection, lesions scattered on almost all the leaves, plant prematurely dried.

#### Phenotypic data analysis

A Mixed linear model was used for analysis of phenotypic data from alpha-lattice design where genotypes, environments, interaction between genotype with environment and interaction with replication and environment were considered as random effects.

$$Y_{ijko} = \mu + g_{i} + l_{j} + r_{kj} + b_{ojk} + e_{ijko}$$

where *Y*_*ijko*_ is phenotypic performance of the *i*th genotype at the *j*th environment in the *k*th replication of the *o*th incomplete block, *μ* was an intercept term, *gi* was the genetic effect of the *i*th genotype, *lj* was the effect of the *j*th environment, *r*_*kj*_ was the effect of the *k*th replication at the *j*th environment, *b*_*ojk*_ was the effect of the *o*th incomplete block in the *k*th replication at the *j*th environment, and *e*_*ijko*_ was the residual. For the CAAM panel and DTMA panel, best linear unbiased predictions (BLUPs) were estimated using Meta-R version 4.1^70^ using anthesis date (AD) parameter as covariate because NCLB scores were significantly correlated to AD. In augmented design trials, BLUPs were estimated across years using linear model for repeated entries and linear model for entries in SAS. Linear model for repeated entry ${Y}_{{k}^{0}jl}=\mu +{\beta }_{j(l)}+{\gamma }_{l}+{\tau }_{{k}^{0}}{+({\tau \gamma )}_{{k}^{0}l}+\varepsilon }_{{k}^{0}jl}$ k^o^ = 1, 2, …, q (repeated entries), j = 1, 2, …, b (blocks), l = 1, 2, …, l (locations) where *β*_*j*(l)_: is the effect of the jth block nested in lth location, *γ*_*l*_: is the effect of the lth location, *τ*_*k*_^*0*^: is the effect of the kth repeated entry, (*τγ*)_*k*_^*0*^_*l*_: is the effect of the interaction between the k^0^th entry and the lth location and the linear model for entries ${Y}_{ijl}=\mu +{\beta }_{j(l)}+{\gamma }_{l}+{\tau }_{i}{+({\tau \gamma )}_{il}+\varepsilon }_{ijl}$. i = 1, 2, …, v (entries), j = 1, 2, …, b (blocks), l = 1, 2, …, l (locations). *β*_*j*(l)_: is the effect of the jth block nested in lth location, *γ*_*l*_: is the effect of the lth location, *τ*_*i*_: is the effect of the ith entry, (*τγ*)_*il*_: is the effect of the interaction between the ith entry and the lth location. Broad- sense heritability (H^2^) of multi-location trials was estimated as H^2^ = σ^2^_g_/( σ^2^_g_ + σ^2^_ge_/*e* + σ^2^_e_/*er*), where σ^2^_g_, σ^2^_ge_ and σ^2^_e_ are the genotypic, genotype-by-environment interaction and error variance components, respectively, and e and r are the number of environments and number of replicates within each environment included in the analysis, respectively. Meta-R version 4.1 was also used in generating descriptive statistics and genetic correlations between the NCLB scores and anthesis date.

#### DNA isolation and genotyping

DNA of all maize lines constituting association mapping panels was isolated from leaf samples of 3–4 weeks old seedlings using the standardised procedure followed by CIMMYT^71^ (CIMMYT 2005). Panels were genotyped at Institute for Genomic Diversity, Cornell University, Ithaca, NY, USA for Single nucleotide polymorphism (SNPs) using genotyping by sequencing method (GBS). The GBS libraries were constructed following the method of Elshire et al.ss^72^, and SNP calling was performed using TASSEL GBS pipeline^73^. Physical co-ordinates of all SNPs were derived from the maize reference genome version B73 AGPV2. The original partially imputed GBS SNP data had 955,690 genotypic data points (SNPs) across all the chromosomes of approximately 22,000 maize lines publicly available through Panzea database (www.panzea.org). For GWAS, filtration criteria of call rate (CR) ≥ 0.7 and minor allele frequency (MAF) ≥ 0.05 were used in all panels, yielding 293,606, 297,437 and 309,608 SNPs for CAAM, DTMA and IMAS panels, respectively. For estimating PCA and kinship matrix, high quality SNPs with filtering criteria of CR ≥ 0.9, MAF ≥ 0.1, and pruned at an *r*^2^ threshold of ≤ 0.5 were used for selecting 64,344 SNPs for CAAM, 69,254 for DTMA and 69,286 for IMAS panel.

#### Principal component, kinship and genome wide linkage disequilibrium analysis

The PCA method described by Price et al*.*^74^ was conducted in all panels using SNP & Variation Suite (SVS) Version_8.6.0 (SVS, Golden Helix, Inc., Bozeman, MT, www. goldenhelix.com). The first three principal components were used to project the possible population stratification among the samples using 3D plot. A kinship matrix was computed from identity-by-state (IBS) distance matrix^75^ as executed in SVS Version_8.6.0. $$IBS distance=\frac{No. of markers IBS2+(0.5 X No. of markers in IBS1)}{Number of non-missing markers}$$. Genome-wide LD was estimated for adjacent high quality SNPs with filtering criteria of CR ≥ 0.9, MAF ≥ 0.1 for CAAM (126,120 SNPs), DTMA (148,013 SNPs) and IMAS (139,061 SNPs) panels respectively, as adjacent-pairwise *r*^2^ values (the squared allele frequency correlations, among alleles at two adjacent SNP markers). For estimation of LD decay across the genome, *r*^2^ values between SNPs were plotted against the physical distances between the SNPs^76^. LD decay plot using non- linear model was plotted in R using ‘*nlin*’ function^77^. Average pairwise distances in which LD decayed at *r*^2^ = 0.2 and *r*^2^ = 0.1 were then estimated based on the model given by Hill & Weir^78^.

#### GWAS and haplotype regression

GWAS was carried out on AD adjusted BLUPs for NCLB resistance employing three methodologies: uncorrected genotypic data only (G-test or naïve model), genotypic data corrected for structure (Q) using 10 principle components (G + Q; general linear model (GLM)) and genotypic data corrected for both structure and kinship (K) (G + Q + K; Single locus mixed linear model (MLM)). G-test and GLM used association test with additive model and MLM used mixed model single locus (EMMAX)^79^ as executed in SVS Version 8.6.0. The mixed association mapping model used was Y = SNP*β + PC*α + K *μ + ε, where Y = response of the dependent variable (NCLB Score), SNP = SNP marker (fixed effects), PC = principal component coordinate from the PCA (fixed effects), K = kinship matrix (random effects), α is the vector of PC, β and μ are the vectors of SNP and K, respectively, and ε is the error. Manhattan plots were plotted using the − log 10 *P* values of all SNPs used in analysis; Q–Q plots were plotted of the observed − log 10 *P* values and the expected − log 10 *P* values to study the genomic inflation. Considering the genome-wide LD between SNPs, the effective number of independent markers was used to obtain the *P* value thresholds. The number of SNPs in linkage equilibrium with each other were estimated at an *r*^2^ threshold of 0.1. A Bonferroni corrected *P* value threshold at α = 1 was used to compute the significant *P* value thresholds^45^ for each panel.

SNPs within the bottom 0.1 percentile of the distribution in GWAS in each study panel were selected for haplotype detection and trait regression in all the three panels. Haplotype frequency estimation was done using the Expectation Maximisation (EM) algorithm with 50 EM iterations^80^, EM convergence tolerance of 0.0001 and a frequency threshold of 0.01. To minimise the historical recombination, haplotype blocks were detected based on the block defining algorithm^81^. Regression analysis was carried out with the haplotypes detected, based on step-wise regression of the NCLB BLUP estimates in all three panels separately with forward elimination at FDR-value cut off of 0.05.

## Supplementary information

Supplementary Tables.
