# Supplementary material for: Genome-wide association studies in tropical maize germplasm reveal novel and known genomic regions for resistance to Northern corn leaf blight
Source: Sci Rep. 2020 Dec 15;10:21949. doi: 10.1038/s41598-020-78928-5 (PMC7738672; doi:10.1038/s41598-020-78928-5)
Supplement: Supplementary file 1 — Supplementary Tables. [file 41598_2020_78928_MOESM1_ESM.docx]

**Genome-wide association studies in tropical maize germplasm reveal novel and known genomic regions for resistance to Northern corn leaf blight**

**Zerka Rashid^1^, Mehrajuddin Sofi^2^, Sharanappa I Harlapur^3^, Rajashekhar M Kachapur^3^, Zahoor Ahmed Dar^4^, Pradeep Kumar Singh^1^, Pervez Haider Zaidi^1^, Bindiganavile Sampath Vivek^1^, Sudha Krishnan Nair*^1^**

**^1^** International Maize and Wheat Improvement Center (CIMMYT), ICRISAT Campus, Patancheru, Greater Hyderabad 502324, Telangana, India

^2^ High Mountain Arid Agricultural Research Institute (HMAARI) Stakna, Leh, SKUAST-Kashmir, 19410, India

^3^ University of Agricultural Sciences. Krishi Nagar Dharwad, 580005, Karnataka, India

^4^ Sher-e-Kashmir University of Agriculture Sciences and Technology (SKUAST), Srinagar 190001, Jammu and Kashmir, India

*Corresponding author: Sudha.nair@cgiar.org

Supplementary table 1: Chromosome-wise average adjacent pair distances between SNPs at which LD decayed at r^2^ = 0.2 and r^2^ =0.1. The distances are given in Kb.

|  | **CAAM** | | **DTMA** | | **IMAS** | |
| --- | --- | --- | --- | --- | --- | --- |
| **Chr.** | **r^2^ = 0.1** | **r^2^ = 0.2** | **r^2^ = 0.1** | **r^2^ = 0.2** | **r^2^ = 0.1** | **r^2^ = 0.2** |
| 1 | 2.5 | 0.87 | 4.97 | 1.72 | 2.71 | 0.95 |
| 2 | 2.24 | 0.78 | 4.21 | 1.46 | 2.69 | 0.94 |
| 3 | 2.8 | 0.98 | 5.14 | 1.78 | 3.13 | 1.09 |
| 4 | 3.85 | 1.34 | 6.32 | 2.19 | 3.7 | 1.27 |
| 5 | 2.34 | 0.81 | 4.54 | 1.57 | 2.52 | 0.88 |
| 6 | 2.27 | 0.79 | 3.2 | 1.11 | 2.02 | 0.7 |
| 7 | 1.79 | 0.62 | 3.24 | 1.12 | 2.04 | 0.71 |
| 8 | 4.69 | 1.63 | 12.81 | 4.43 | 5.24 | 1.83 |
| 9 | 2.28 | 0.79 | 4.44 | 1.54 | 2.6 | 0.91 |
| 10 | 3.41 | 1.19 | 7.24 | 2.51 | 3.47 | 1.21 |

Supplementary table 2: Haplotype regression analysis for NCLB resistance in CAAM panel with FDR value cut off ≤0.05.

| **Haplotype blocks** | **Chr** | **Markers Used** | **# Haplotypes** | **P-Value** | **R^2^ (%)** | **FDR** | **Favorable alleles** |
| --- | --- | --- | --- | --- | --- | --- | --- |
| Hap_1.1C | 1 | S1_12486888, S1_12486904 | 2 | 1.09E-06 | 6.378 | 4.08E-05 | CC |
| Hap_1.2C | 1 | S1_90892964, S1_90892966, S1_90892967, S1_90892970 | 2 | 0.00355 | 2.789 | 0.020925 | TTTG |
| Hap_2.1C | 2 | S2_44199513, S2_44199556 | 2 | 5.10E-07 | 6.722 | 2.86E-05 | CG |
| Hap_2.2C | 2 | S2_53938282, S2_53938363 | 2 | 0.000928 | 3.572 | 0.008661 | CC |
| Hap_2.3C | 2 | S2_158673333, S2_158673408, S2_158673422, S2_158673429, S2_158674708 | 3 | 0.005127 | 2.198 | 0.028709 | CGCGT |
| Hap_2.4C | 2 | S2_163567715, S2_163567727 | 2 | 0.008743 | 1.981 | 0.04663 | CT |
| Hap_2.5C | 2 | S2_208538471, S2_211029206 | 2 | 0.001659 | 2.889 | 0.012385 | TA |
| Hap_4.1C | 4 | S4_11081178, S4_11081212 | 2 | 1.15E-05 | 6.792 | 0.000259 | AA |
| Hap_4.2C | 4 | S4_66558784, S4_66558850, S4_66558853, S4_66558873 | 3 | 0.001331 | 2.904 | 0.01065 | GAGC |
| Hap_4.3C | 4 | S4_136671077, S4_136671078, S4_136671079, S4_136671091 | 2 | 0.002837 | 2.921 | 0.017651 | GCTT |
| Hap_4.4C | 4 | S4_236938491, S4_236938493, S4_236938494 | 2 | 0.002117 | 2.608 | 0.013944 | TTC |
| Hap_5.1C | 5 | S5_15773162, S5_15773163 | 2 | 7.93E-06 | 6.092 | 0.000222 | GC |
| Hap_5.2C | 5 | S5_178352064, S5_178352380 | 2 | 0.000328 | 5.267 | 0.00367 | AT |
| Hap_5.3C | 5 | S5_204091738, S5_204091741 | 2 | 6.67E-05 | 4.576 | 0.00083 | GA |
| Hap_6C | 6 | S6_156321351, S6_156321360, S6_156931308 | 2 | 0.001878 | 2.891 | 0.013147 | TTG |
| Hap_8.1C | 8 | S8_95422954, S8_95422964, S8_95422973 | 2 | 2.89E-05 | 6.445 | 0.000405 | CAT |
| Hap_8.2C | 8 | S8_119494934, S8_119494935, S8_119494937, S8_119494938 | 2 | 2.41E-05 | 5.268 | 0.000386 | TACT |
| Hap_9.1C | 9 | S9_57188661, S9_57188668 | 2 | 1.52E-07 | 8.468 | 1.70E-05 | CG |
| Hap_9.2C | 9 | S9_99293028, S9_99293080 | 3 | 0.000392 | 4.476 | 0.003991 | GA |
| Hap_10.1C | 10 | S10_18140579, S10_18140580, S10_18140584, S10_18140587 | 2 | 0.001221 | 3.500 | 0.010521 | GCCC |
| Hap_10.2C | 10 | S10_109345864, S10_109345865, S10_109345872 | 3 | 1.47E-05 | 5.090 | 0.000274 | ATA |

Supplementary table 3: Haplotype regression analysis for NCLB resistance in DTMA panel with FDR value cut off ≤0.05

| **Haplotype block** | **Chr** | **Markers Used** | **# Haplotypes** | **P-Value** | **R^2^ (%)** | **FDR** | **Favorable alleles** |
| --- | --- | --- | --- | --- | --- | --- | --- |
| Hap_1.1D | 1 | S1_83522387, S1_83522395 | 2 | 0.015678 | 2.65 | 0.045025 | CC |
| Hap_1.2D | 1 | S1_90892964, S1_90892966, S1_90892967, S1_90892970 | 2 | 2.43E-05 | 8.71 | 0.000247 | TTTG |
| Hap_1.3D | 1 | S1_230882239, S1_230882260, S1_230882354, S1_230882360, S1_230882374 | 2 | 0.001093 | 5.33 | 0.00583 | CTATG |
| Hap_2.1D | 2 | S2_4193026, S2_4193164 | 2 | 0.002481 | 4.32 | 0.01158 | CG |
| Hap_2.2D | 2 | S2_6597154, S2_6597155 | 2 | 5.96E-06 | 9.63 | 7.42E-05 | CC |
| Hap_2.3D | 2 | S2_23669574, S2_23669594 | 2 | 2.11E-07 | 12.57 | 1.18E-05 | CC |
| Hap_2.4D | 2 | S2_158673333, S2_158673408, S2_158673422, S2_158673429, S2_158674708 | 3 | 0.004642 | 3.59 | 0.01677 | CGTGA |
| Hap_2.5D | 2 | S2_180519190, S2_180519195, S2_180519197 | 2 | 6.70E-05 | 9.78 | 0.0005 | GCA |
| Hap_2.6D | 2 | S2_208538471, S2_211029206 | 2 | 0.013012 | 2.81 | 0.039387 | TA |
| Hap_3.1D | 3 | S3_19558054, S3_19558077 | 2 | 0.005165 | 3.58 | 0.018079 | AA |
| Hap_3.2D | 3 | S3_166449259, S3_166449260, S3_166449261, S3_166449975 | 3 | 2.97E-07 | 13.90 | 1.11E-05 | GTCC |
| Hap_3.3D | 3 | S3_176553663, S3_176553666 | 2 | 3.67E-06 | 9.38 | 6.84E-05 | TC |
| Hap_3.4D | 3 | S3_218772089, S3_218772093 | 3 | 0.003133 | 4.60 | 0.012532 | TC |
| Hap_4.1D | 4 | S4_11081178, S4_11081212 | 2 | 0.00407 | 4.89 | 0.015719 | AA |
| Hap_4.2D | 4 | S4_66559053, S4_66559106 | 3 | 6.45E-06 | 9.74 | 7.23E-05 | GC |
| Hap_4.3D | 4 | S4_66617559, S4_66617679 | 2 | 1.33E-07 | 13.08 | 1.49E-05 | TC |
| Hap_4.4D | 4 | S4_118899746, S4_118899762, S4_118899779, S4_118899995, S4_118900059, S4_118900271 | 2 | 0.004354 | 3.74 | 0.016256 | CGGCGC |
| Hap_4.5D | 4 | S4_136671077, S4_136671078, S4_136671079, S4_136671091 | 2 | 3.67E-05 | 8.47 | 0.000342 | GCTT |
| Hap_4.6D | 4 | S4_136960284, S4_136960290 | 2 | 0.000112 | 6.66 | 0.000785 | CC |
| Hap_4.7D | 4 | S4_236938491, S4_236938493, S4_236938494 | 2 | 0.000433 | 5.69 | 0.002695 | CCT |
| Hap_5D | 5 | S5_204091738, S5_204091741 | 2 | 0.002912 | 3.99 | 0.013044 | GA |
| Hap_6D | 6 | S6_155756665, S6_155756666, S6_155756667, S6_155756669, S6_155756671, S6_155756673, S6_155756674, S6_155756678 | 2 | 0.00313 | 4.53 | 0.012982 | AAAACAGA |
| Hap_7.1D | 7 | S7_110058157, S7_110058160 | 2 | 4.96E-05 | 7.59 | 0.000397 | AC |
| Hap_7.2D | 7 | S7_110282281, S7_110282311, S7_110282335, S7_110282502, S7_110282525 | 3 | 4.62E-07 | 13.13 | 1.29E-05 | CCGCA |
| Hap_7.3D | 7 | S7_130426186, S7_130426201 | 2 | 0.005768 | 4.11 | 0.019575 | CC |
| Hap_7.4D | 7 | S7_168578729, S7_168578731, S7_168578733 | 2 | 0.001535 | 4.59 | 0.007816 | AGC |
| Hap_8.1D | 8 | S8_21448074, S8_21448086, S8_21448088, S8_21448089 | 2 | 0.00933 | 3.06 | 0.029857 | CAGA |
| Hap_8.2D | 8 | S8_105321803, S8_105321804, S8_105321807, S8_105321808, S8_105321834 | 2 | 0.001959 | 4.39 | 0.009542 | AGCAA |
| Hap_8.3D | 8 | S8_131534491, S8_131534569 | 3 | 0.000479 | 5.43 | 0.002824 | CA |
| Hap_8.4D | 8 | S8_144174845, S8_144649760, S8_144649825 | 3 | 4.81E-06 | 9.72 | 7.70E-05 | GGC |
| Hap_8.5D | 8 | S8_167996301, S8_167996304 | 2 | 0.003013 | 4.21 | 0.012979 | CA |
| Hap_9.1D | 9 | S9_14906566, S9_14906568, S9_14906569, S9_14906572, S9_14906573, S9_14906574, S9_14906575, S9_14906576, S9_14906577, S9_14906578 | 2 | 0.015371 | 3.78 | 0.045304 | AATGATTTTT |
| Hap_9.2D | 9 | S9_19393290, S9_19393702 | 2 | 4.40E-05 | 7.55 | 0.000379 | AC |
| Hap_9.3D | 9 | S9_99293028, S9_99293080 | 3 | 0.000277 | 6.47 | 0.001828 | AC |
| Hap_9.4D | 9 | S9_113423624, S9_113423632 | 2 | 0.000746 | 5.79 | 0.004177 | AG |
| Hap_9.5D | 9 | S9_151289456, S9_151289599 | 2 | 3.59E-06 | 10.40 | 8.05E-05 | CC |
| Hap_10.1D | 10 | S10_1422632, S10_1422674 | 2 | 0.005862 | 3.70 | 0.01931 | AA |
| Hap_10.2D | 10 | S10_88268774, S10_88268837 | 2 | 5.51E-06 | 9.43 | 7.72E-05 | GG |
| Hap_10.3D | 10 | S10_109345864, S10_109345865, S10_109345872 | 3 | 0.012477 | 2.99 | 0.038816 | CCT |

Supplementary table 4: Haplotype regression analysis for NCLB resistance in IMAS panel with FDR value cut off ≤0.05

| **Haplotype block** | **Chr** | **Markers Used** | **# Haplotypes** | **P-Value** | **R2(%)** | **FDR Value** | **Favorable alleles** |
| --- | --- | --- | --- | --- | --- | --- | --- |
| Hap_1.1I | 1 | S1_65745651, S1_65745682, S1_65745684, S1_65745685 | 2 | 1.18E-06 | 6.815 | 2.20E-05 | CGTA |
| Hap_1.2I | 1 | S1_105463521, S1_105463522, S1_105463557 | 4 | 4.02E-06 | 8.043 | 5.63E-05 | ATT |
| Hap_1.3I | 1 | S1_107680879, S1_107680880, S1_107680886, S1_107680924, S1_107791749 | 4 | 4.92E-05 | 5.974 | 0.000424 | GTCGG |
| Hap_1.4I | 1 | S1_161617710, S1_161617735 | 2 | 8.12E-05 | 4.759 | 0.000535 | GG |
| Hap_1.5I | 1 | S1_211324372, S1_211324374, S1_211324396 | 2 | 2.29E-06 | 7.185 | 3.67E-05 | ATT |
| Hap_1.6I | 1 | S1_230882239, S1_230882260, S1_230882354, S1_230882360, S1_230882374 | 3 | 0.001229 | 3.544 | 0.004589 | GCGAC |
| Hap_1.7I | 1 | S1_293793763, S1_293793767 | 2 | 0.014192 | 1.888 | 0.044153 | GG |
| Hap_2.1I | 2 | S2_2364758, S2_2364759 | 2 | 2.17E-05 | 5.342 | 0.00027 | AT |
| Hap_2.2I | 2 | S2_141154138, S2_141160057 | 3 | 0.000262 | 4.789 | 0.001222 | AA |
| Hap_2.3I | 2 | S2_207379402, S2_207379404, S2_207379412, S2_207379413, S2_207379414 | 2 | 0.000318 | 4.264 | 0.001423 | GTGGG |
| Hap_3.1I | 3 | S3_6432571, S3_6432572, S3_6432573, S3_6432575 | 2 | 7.08E-05 | 4.807 | 0.000529 | TCAT |
| Hap_3.2I | 3 | S3_129163060, S3_129163082 | 2 | 0.000104 | 4.522 | 0.000582 | TC |
| Hap_3.3I | 3 | S3_177447542, S3_177447792, S3_177447794 | 2 | 0.000359 | 4.057 | 0.001547 | TTC |
| Hap_3.4I | 3 | S3_179513253, S3_179513254 | 2 | 0.000216 | 4.021 | 0.001098 | TC |
| Hap_3.5I | 3 | S3_230526186, S3_230526187 | 2 | 0.016847 | 2.023 | 0.049654 | CT |
| Hap_4.1I | 4 | S4_66558784, S4_66558850, S4_66558853, S4_66558873 | 2 | 7.97E-07 | 7.277 | 1.79E-05 | ATAT |
| Hap_4.2I | 4 | S4_208245677, S4_208245679, S4_208245995 | 3 | 9.86E-08 | 8.506 | 3.68E-06 | CCG |
| Hap_5I | 5 | S5_204091738, S5_204091741 | 2 | 1.40E-08 | 9.123 | 7.82E-07 | GA |
| Hap_6I | 6 | S6_114920296, S6_114920312 | 2 | 0.000101 | 4.409 | 0.000592 | GT |
| Hap_7.1I | 7 | S7_82324473, S7_82324504 | 3 | 8.44E-05 | 4.752 | 0.000525 | TC |
| Hap_7.2I | 7 | S7_107693777, S7_107693791, S7_107693801 | 2 | 2.21E-05 | 5.703 | 0.000248 | CGG |
| Hap_8.1I | 8 | S8_95422954, S8_95422964, S8_95422973 | 2 | 0.011805 | 2.342 | 0.037776 | CAT |
| Hap_8.2I | 8 | S8_105321803, S8_105321804, S8_105321807, S8_105321808, S8_105321834 | 3 | 0.000802 | 3.445 | 0.003096 | TAAGG |
| Hap_8.3I | 8 | S8_106236368, S8_106236376 | 3 | 0.000157 | 4.181 | 0.000838 | GG |
| Hap_8.4I | 8 | S8_131534491, S8_131534569 | 4 | 0.015592 | 1.713 | 0.047197 | CA |
| Hap_8.5I | 8 | S8_157985530, S8_157986156, S8_157986163, S8_157987471, S8_157987565, S8_157987595, S8_157987611 | 3 | 4.48E-09 | 11.503 | 5.02E-07 | ACATTTT |
| Hap_8.6I | 8 | S8_171402085, S8_171402118 | 2 | 0.001621 | 3.542 | 0.005857 | TC |
| Hap_9.1I | 9 | S9_14906566, S9_14906568, S9_14906569, S9_14906572, S9_14906573, S9_14906574, S9_14906575, S9_14906576, S9_14906577, S9_14906578 | 2 | 0.00023 | 4.892 | 0.001121 | AATGATTTTT |
| Hap_9.2I | 9 | S9_25926625, S9_25926626, S9_25926627 | 2 | 2.56E-05 | 5.160 | 0.00026 | ACA |
| Hap_10.1I | 10 | S10_11897599, S10_11897605, S10_11897606 | 2 | 0.000454 | 4.244 | 0.001884 | ACT |
| Hap_10.2I | 10 | S10_18140579, S10_18140580, S10_18140584, S10_18140587 | 2 | 0.010931 | 2.307 | 0.036007 | GCCC |
| Hap_10.3I | 10 | S10_62371841, S10_62371863, S10_62371868, S10_62371869 | 2 | 6.93E-07 | 7.019 | 1.94E-05 | TCGT |
| Hap_10.4I | 10 | S10_62943704, S10_62943753, S10_63031407, S10_63031432, S10_63031478 | 3 | 4.34E-05 | 5.258 | 0.000405 | CATTT |
| Hap_10.5I | 10 | S10_88268774, S10_88268837 | 3 | 0.009943 | 2.009 | 0.033747 | GG |
| Hap_10.6I | 10 | S10_92024066, S10_92024068, S10_92024071, S10_92024072, S10_92024082, S10_92024084, S10_92024085 | 2 | 0.000766 | 3.460 | 0.003064 | CCGGGCT |
| Hap_10.7I | 10 | S10_117620784, S10_117620808 | 3 | 0.002484 | 2.877 | 0.008694 | GG |
| Hap_10.8I | 10 | S10_141950978, S10_141950983 | 2 | 5.01E-05 | 5.694 | 0.0004 | GT |
| Hap_10.9I | 10 | S10_147820379, S10_147820381 | 2 | 7.75E-05 | 5.064 | 0.000543 | AC |
